# Ten simple rules for organizations to support research data sharing

**DOI:** 10.1371/journal.pcbi.1011136

**Published:** 2023-06-15

**Authors:** Robin Champieux, Anthony Solomonides, Marisa Conte, Svetlana Rojevsky, Jimmy Phuong, David A. Dorr, Elizabeth Zampino, Adam Wilcox, Matthew B. Carson, Kristi Holmes

**Affiliations:** 1 OHSU Library, Oregon Health and Science University, Portland, Oregon, United States of America; 2 Research Institute, NorthShore University Health System, Evanston, Illinois, United States of America; 3 Department of Learning Health Sciences, University of Michigan Medical School, Ann Arbor, Michigan, United States of America; 4 Tufts Clinical and Translational Science Institute, Tufts University, Boston, Massachusetts, United States of America; 5 UW Medicine Research IT, University of Washington, Seattle, Washington, United States of America; 6 Harborview Injury Prevention and Research Center, Seattle, Washington, United States of America; 7 Department of Medical Informatics & Clinical Epidemiology, OHSU, Portland, Oregon, United States of America; 8 Division of Biomedical and Health Informatics, University of Washington, Seattle, Washington, United States of America; 9 Institute for Informatics, Washington University in St. Louis School of Medicine, St. Louis, Missouri, United States of America; 10 Department of Medicine, Washington University in St. Louis School of Medicine, St. Louis, Missouri, United States of America; 11 Galter Health Sciences Library and Learning Center, Northwestern University Feinberg School of Medicine, Chicago, Illinois, United States of America; 12 Department of Preventive Medicine, Northwestern University Feinberg School of Medicine, Chicago, Illinois, United States of America; Carnegie Mellon University, UNITED STATES

This is a *PLOS Computational Biology* Methods paper.

## Introduction

Scientific discovery depends on access to data and the knowledge this data makes possible. Research data sharing is increasingly recognized as a priority for organizations to support the successful conduct of research. The National Institutes of Health states, “data sharing enables researchers to rigorously test the validity of research findings, strengthen analyses through combined datasets, reuse hard-to-generate data, and explore new frontiers of discovery” [1]. Conversely, in the absence of data sharing, there are increased risks related to the robustness, rigor, and replicability of results, and the potential of valuable data is diminished. For these reasons and more, institutional data sharing capacity is a critical topic for organizations to scrutinize, discuss, and advance.

Advocacy and support for data sharing are often discussed with an emphasis on understanding and supporting the practices of individual investigators or scientific communities [2,3]. However, a researcher’s ability to successfully engage in and benefit from sound data sharing depends on their organizational setting and, specifically, the organization’s data sharing capacity. For example, sharing data is easier and more equitable when organizational processes and procedures are established and documented, and research workforce members can access centralized training and infrastructure resources.

The effect of how an organization approaches and supports data sharing extends beyond the success of its investigators. Institutions that share data can participate in innovative large-scale initiatives and pursue new funding opportunities. Universities that contribute to creating data sharing innovations, including best practices and ethical guidelines, can realize more meaningful impacts for the communities they support and serve. Organizations that intentionally invest in research data sharing are better prepared to comply with the evolving policy landscape. Thus, an institution’s data sharing capacity is both an influencer and an indicator of scientific success.

This paper facilitates a general discussion for organizations about data sharing capacity. We introduce the utility of maturity models (Rule 1), discuss the importance of understanding users and use cases (Rule 2), and emphasize the value of communication and collaboration (Rule 3). To urge the recognition and examination of a range of key facets, we also present rules highlighting 7 organizational elements that enable or impede data sharing (Rules 4 to 10) based on a recent maturity model [4]. Overall, we aim to call attention to the various systems, technologies, policies, practices, values, and people that impact an organization’s data sharing effectiveness. These Rules can guide an organization through key strategic and practical research data activities, from the assembly of a team and needs assessment across key facets to the development or augmentation of formal structures, resources, and services needed for the organization to be able to facilitate data sharing successfully. While this paper has been written for organizations, it is intended to reflect the experiences and needs of local research communities. Therefore, for several Rules, we have included key questions research workforce members may ask, which organizations should be prepared to answer with actionable information.

## Rule 1: Use a maturity model to understand organizational qualities that impact data sharing

Organizations need to understand their baseline capabilities for data sharing, and a maturity model can facilitate this assessment. A maturity model is a framework for describing and evaluating the processes, structures, technology, culture, and people associated with and enabling growing effectiveness in an area of focus, such as data management or research IT [5,6]. Maturity models are used to identify strengths and weaknesses and to generate improvement plans. They describe the factors or domains associated with an area of focus along a scale of increasing capacity. Thus, any organization can identify itself in a model and grade its current capabilities in each domain. As many institutions are just beginning to plan for data sharing at an organizational scale, this feature of maturity models is especially useful.

**Fig 1:**
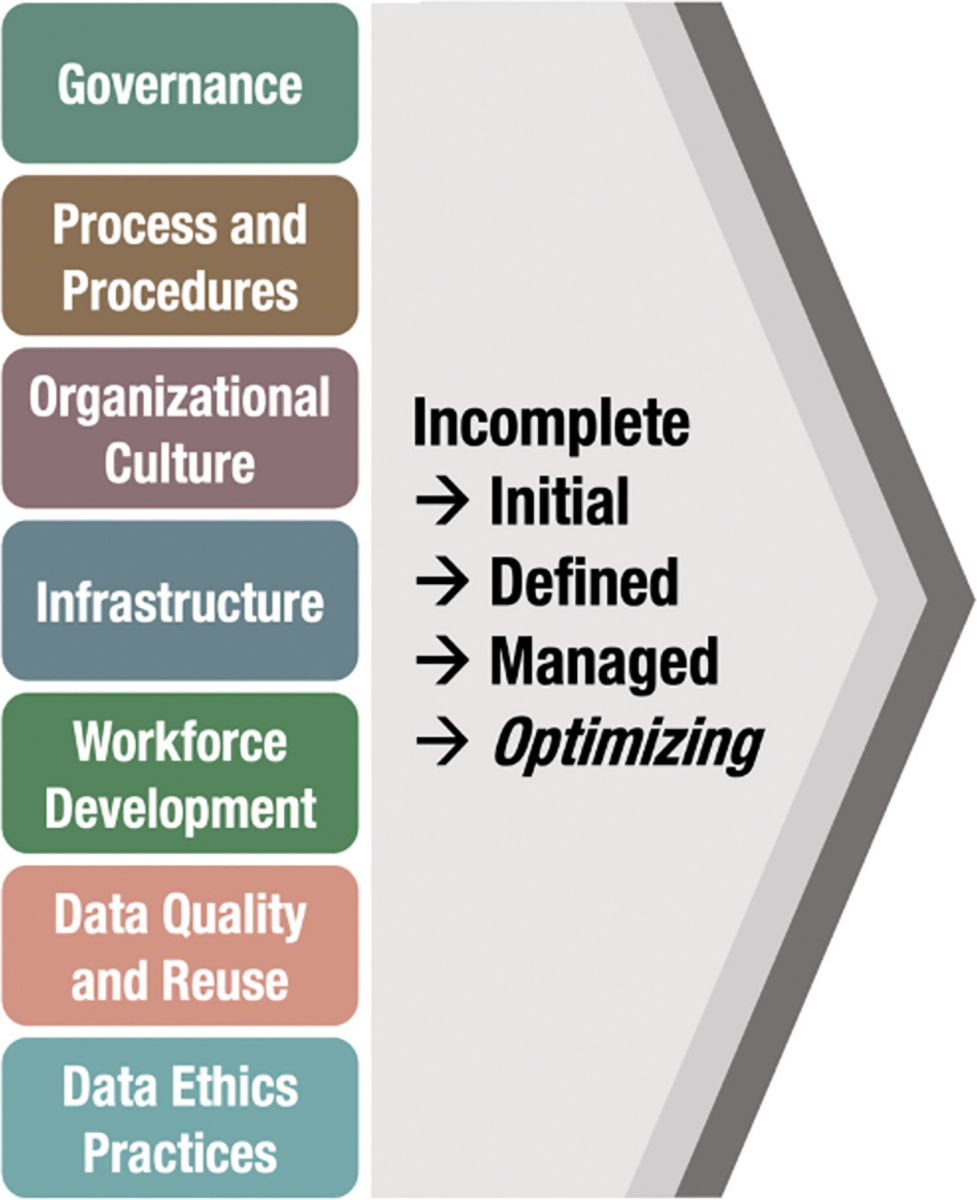
Schematic depiction of the Research Data Sharing Capacity Maturity Model.

“Research Data Sharing: A Maturity Model for Organizational Capacity” (Fig 1) describes 7 interdependent domains: governance, process and procedures, organizational culture, infrastructure, workforce development, data quality and reuse, and data ethics practices [4]. The levels for each progress from Level 1, characterized by a lack of resources or focused action, to Level 5, characterized by stable and continuously improved capabilities. While thorough, this maturity model is not intended to be prescriptive. Our goal is to be inclusive of the variety of organizations interested in sharing or using shared data while drawing attention to the processes, structures, technology, cultural elements, and people essential to doing so. Organizations can explore each domain from levels 1 to 5 to understand what increased capability looks like and to identify their current maturity, which is likely to differ by area. “Research Data Sharing: A Maturity Model for Organizational Capacity” can also serve as a tool to guide a gap analysis, helping organizations identify specific resource investments and interventions to increase their data sharing performance.

## Rule 2: Leverage personas and user stories to better understand local motivations and challenges

A thorough stakeholder assessment is required to advance data sharing on an organizational level. User personas are a helpful tool for identifying and understanding the range of roles and expertise involved with data sharing. A persona is a composite user profile that describes archetypal attributes, such as motivations, pain points, professional development, and technical attitudes. For example, Personas for the Translational Workforce include a range of roles relevant to data sharing, such as data analyst, biostatistician, librarian, IRB (Institutional Review Board) manager, and research administrator [7]. The intentional consideration of workforce roles that personas facilitate can help organizations assess and implement training, resources, and other support for data sharing.

User stories are another effective device for building actionable knowledge about the people and activities involved in data sharing and responding to their needs. User stories typically follow a standard format, such as the Connextra template [8], which provides a simple framework for writing concise prose to communicate a user story: **As a *___* [who/persona], I want *___* [what/requirement], so that *___* [why/outcome]**. Incorporating user stories helps to provide additional context about end users and their perspectives. Personas and user stories can be created or updated as needed to frame and prioritize the investments required to build institutional capacity for data sharing in a locally relevant and inclusive manner (Table 1).

**Table 1:** Data sharing user stories.

Data sharing user stories
◾ As a **RESEARCHER** with data resulting from a grant-funded project, I want to comply with data sharing requirements from my funder so that I can continue my research. ◾ As a **DATA CURATOR**, I need professional development opportunities and reference materials for dataset curation to ensure that I can use best data practices. ◾ As a **RESEARCH ADMINISTRATOR**, I need to make sure that research data management and sharing plans align with funder requirements. ◾ As an **IT PROFESSIONAL**, I need to understand federal system requirements and how our infrastructure can best meet the needs of researchers generating sensitive or private data. ◾ As a **LIBRARIAN**, I want to learn about data sharing policies and workflows to support researchers. ◾ As **RESEARCH COMPUTING SUPPORT**, I need to stay up-to-date on the latest requirements and resources that can support our campus research data sharing workflows. ◾ As an **ORGANIZATIONAL LEADER**, I want to know what resources and services are needed so that we can budget and plan for them. ◾ As a **REPOSITORY MANAGER**, I want to understand priorities related to data deposit, particularly where certain types of data should be deposited and how to meet best practices for making research discoverable, while safeguarding privacy or security considerations. ◾ As a **COMMUNITY-BASED PARTNER** of the cancer center, I want to have access to the research discoveries from the center so that I can track recent results in areas that impact my family and community. ◾ As a **MEMBER OF THE PUBLIC**, I want to have access to the results of research that my tax dollars fund so that there is increased accountability and transparency in research.

## Rule 3: Foster communication and collaboration to advance data sharing

Effective communication and collaboration are essential for modern research. Given the institution-wide scope of systems, technologies, policies, practices, values, and people involved, investment in communication and collaboration nurtures organizational data sharing capacity. The field of team science provides a practical foundation for this work and includes a wide range of resources. For example, the Center for Research, Excellence and Diversity in Team Science (CREDITS) has compiled a collection of team science resources in the Inclusive Collaboration Toolkit [9]. The Toolkit provides knowledge and tools to maximize the efficiency and effectiveness of inclusive team science activities. COALESCE is a free online team science training tool that provides short videos and activities covering topics such as collaborative decision-making with communities [10]. These and other resources [11] from the field of team science can help institutions understand the collaborative contexts in which data is generated and shared, foster effective communication and collaboration, and build trust among the many partners involved in developing and delivering resources and services for the organization.

Rules 1 through 3 describe foundational approaches and tools to help organizations understand and grow their data sharing capacity, particularly concerning the factors discussed in Rules 4 to 10. Each rule is annotated with sample questions research workforce members will likely ask their organization (Box 1–7).

## Rule 4: Develop governance structures and empower leaders and stakeholders to drive policy

Governance establishes an organization’s action in data sharing through the creation and communication of policies that define expectations, provide consistent guidance for routine considerations, and reflect its unique needs and capabilities. Ideally, a governance structure should include active participation from accountable leaders and internal and external parties across the data lifecycle [6]. This may include chief research officers, investigators from diverse disciplines, librarians, technology transfer specialists, regulatory managers, research participants and partners, and leaders with relevant technical expertise. Inclusive and effective governance reduces ad hoc decision-making, duplicative effort, and mistakes that often result from it, making data sharing easier and more efficient for researchers. Inclusive and effective governance also reduces institutional risk, such as that related to the appropriate sharing of certain types of data (e.g., sensitive data or data perceived to have commercial potential), and the organization is better positioned to respond to new opportunities and requirements quickly and flexibly.

An effective approach to establishing a governance structure is to develop a RACI matrix, where roles and their levels of authority are mapped to specific functions. The 4 levels are described as **R**esponsible (who does the work), **A**ccountable (who ensures that the work is done as specified), **C**onsulted (who influences how the work is done), and **I**nformed (who needs to know, even if they have no direct influence over the content of the work). RACI provides clarity to ensure all functions are covered, that roles are not overloaded or in conflict, and that there is a path from consultation and responsibility to accountability.

Increasingly, organizational governance structures include comprehensive and collaborative research data services units that address oversight and include contributions from key units and stewards on campus, such as Offices for Research, Information Technology, Research Computing, and Libraries. Notable examples include Cornell University’s Research Data Management Service Group (RDMSG) and Research Data Management @Harvard [12,13].

**Box 1. Governance research workforce questions**

- What units oversee data sharing procedures and how are decisions made?
- Is there documentation I can use to inform my lab about the organization’s data sharing requirements?
- Where can I go to ask questions about data sharing?
- What governance structures are in place to oversee private or sensitive data?

## Rule 5: Provide clear data sharing workflows and step-by-step procedures

Effective, ethical, and sustainable data sharing relies on transparent workflows and procedures that address technical, social, and governance considerations. Building and maintaining these processes requires a fully informed understanding of an organization’s data sharing landscape and overall strategy, including the key roles and needs of data owners, stewards, service providers, and reuse actors [14]. Different data types will require specific approaches, and well-documented workflows and procedures should provide clear pathways for engaging with operational, technical, and regulatory requirements.

Successful data sharing requires defining and aligning activities and developing clear action plans to support these activities. This planning process accounts for temporal considerations and dependencies and often reveals iterative approval processes (e.g., Institutional Review Board approvals and data use agreements). Organizations should provide clear guidance to inform compliance with specific policies and consider developing a handbook of standard operating procedures (SOP) to support the staff and leaders responsible for transparent and routine centralized support. This handbook could include such processes as communication and approved messaging, roles and responsibilities of collaborating units, best practice workflows for common activities, processes for sensitive data or long-term preservation, and more. The resulting documentation allows research teams and institutional service providers to manage critical and time-sensitive steps, such as those related to de-identification or transferring large volumes of data in a transparent and dependable manner.

**Box 2. Procedures research workforce questions**

- Where do I sign up for updates about data sharing?
- Where can I learn more about the data sharing best practices I should follow?
- How do I ensure I am prepared to comply with my funders’ policies?
- I need someone to review the Data Management and Sharing Plan before I submit my grant; who can help?

## Rule 6: Nurture and codify institutional data sharing values

Institution-wide engagement with sustainable and productive data sharing is dependent on and expressed by an organization’s values. We consider an institution’s organizational culture about data sharing to encompass how leaders and researchers generally interpret data sharing, how its reward systems express these attitudes, and how it treats decisions for engaging with new data sharing opportunities and best practices.

Currently, few research institutions wholly embody the vision of the most mature expression of this domain, wherein: an institution highly and publicly values data sharing; researchers are recognized for sharing through processes like promotion and tenure; ways of identifying and measuring data sharing contributions are continuously considered; and resources for engaging with new data sharing opportunities and best practices are regarded as necessary investments. However, we are inspired by previous work describing the importance of culture in facilitating data sharing and open science [15,16].

Organizations embarking on this journey do not need to start from scratch. Many parties, including funders, regulatory bodies, libraries, and scientific communities have developed guidelines and imagined innovative models for building a culture of data sharing. For example, Piwowar and colleagues propose that department chairs explicitly encourage faculty to monitor how and why their data is reused so that it can be described and rewarded during hiring and promotion decisions [17]. Additionally, they recommend that introductory research curricula include learning outcomes and instruction related to data sharing. Wood-Charlson and colleagues advocate for celebrating FAIR (Findable Accessible Interoperable Reusable) and reproducible data and offer several scalable suggestions, such as using FAIR checklists when students and postdocs depart a lab or research program [18]. Moreover, the recently announced Open Global Data Citation Corpus will develop a trusted and openly available aggregate of all references to research data from diverse sources [19]. This powerful infrastructure for open data metrics will enable monitoring of impact, inform future funding, improve the dissemination of research, and help elucidate and credit data sharing and reuse.

**Box 3. Values research workforce questions**

- How does the organization credit researchers who follow FAIR Practices?
- How are data sharing and data reuse recognized in hiring and promotion decisions?
- Where can I learn about and connect with other researchers generating multimodal data?

## Rule 7: Provide infrastructure to support data sharing

Institutional support for data sharing must be met with continuous investment in the development of technical and social infrastructure. Without this, an unmanageable number of bespoke solutions for tasks associated with data sharing will be created across the lifespan of a research project, from inception to curation, dissemination, and archiving. While this approach may help researchers satisfy immediate needs or requirements, it is both inefficient and unsustainable. It creates a patchwork of strategies, services, and tools that are impossible to maintain at scale. Additionally, a lack of infrastructure makes meaningful governance (Rule 4) difficult.

Examples of infrastructure include tools for data collection (e.g., electronic health records), storage for both working and archival data (e.g., research data warehouses, domain and generalist repositories), and dissemination (e.g., institutional repositories). Information technology departments, integrity offices, core research facilities, and libraries, among others, can advise on necessary considerations, such as security and privacy, interoperability, and FAIR data requirements. Social infrastructure includes resources to support training, develop user communities, and anticipate emerging needs. Ultimately, mature infrastructure at the institutional level requires buy-in from financial and policy decision-makers, acceptance from end users, and feedback loops that direct continuous improvement.

Infrastructure capacity differs by organization, and investment will reflect local priorities and means. As noted above, organizations can use “Research Data Sharing: A Maturity Model for Organizational Capacity” to inventory, assess, and prioritize existing and desired resources, services, and expertise. Shared and reusable data sharing infrastructure allows institutions to leverage economies of scale and access infrastructure that would otherwise be out of reach or peripheral compared to other needs. For example, institutions can utilize open-source repository software, such as Harvard Dataverse [20] or InvenioRDM [21], which powers Zenodo [22], or consider contracting with a generalist repository service provider, such as Dryad [23]. The benefits of these and other data sharing shared infrastructures extend beyond cost savings. The community-based governance and management that underpin them drive innovation and compliance with emerging practices that are otherwise hard to achieve and resource.

**Box 4. Infrastructure research workforce questions**

- What long-term data storage options are available at the organization, and how much do they cost?
- Are there resources available that can help me develop a data management plan?
- Is there someone who can help me with the de-identification of my dataset?
- How can I share large datasets with my collaborators?

## Rule 8: Leverage training and resources to support a wide-ranging data workforce

Data sharing requires a range of activities and skills beyond a single investigator uploading a dataset to a repository. The people involved in data sharing need access to training that supports their specific roles and addresses a range of knowledge levels.

Fortunately, many communities have been proactive about developing and disseminating training infrastructure for data sharing that institutions can and should leverage.

On the local level, campus libraries often lead data sharing training efforts, frequently collaborating with campus IT and research offices. Robust local training infrastructure allows workforce members to tap into guidance that reflects institutional policies, procedures, and resources. Moreover, localized training events and forums (e.g., collaborative documentation, email lists, communication platforms) can foster community and collaboration. Organizations have invested in developing and sustaining a broad array of resources at the national, international, and disciplinary levels. Notable examples include the Carpentries, a global community that teaches data and computational skills for conducting efficient, open, and reproducible research [24]; the FASEB DataWorks! program, an initiative that promotes best practices in data sharing and reuse to advance human health [25]; and the Network of the National Library of Medicine (NNLM) National Center for Data Service (NCDS), which provides training to increase data science capacity among information professionals [26]. These initiatives and others greatly expand an institution’s and our collective knowledge-building capacity for data sharing.

**Box 5. Training research workforce questions**

- What data sharing training does the organization provide?
- Can I earn continuing education credits or certifications when participating in data sharing training?
- Does my department provide professional development funds for data sharing training?
- Can students participate in data sharing training?

## Rule 9: Commit to data quality and reuse standards and best practices

To facilitate scientific discovery and position its research community for success, organizations should actively commit to and support best practices that facilitate reuse to advance data sharing. These include but are not limited to the FAIR Principles, which have guided best practices for sharing machine-readable data since their publication in 2016, and a broad spectrum of standards that govern data structure, content, and description [27]. For example, biomedical researchers can use common data elements to systematize data collection and consistently represent disease diagnoses, medications, procedures, and laboratory tests [28]. Employing metadata best practices, such as the DataCite metadata schema [29], ensures broad discoverability of data and metadata through discovery tools such as Google Dataset Search [30] and DataCite Commons [31].

There are many opportunities for institutions to leverage governance, infrastructure, and training to encourage researchers’ adoption and use of best practices. For instance, they can create and maintain information resources that point to applicable standards and offer training to incorporate them within existing research infrastructure (e.g., electronic laboratory notebooks). Institutions should invest in data curation capacity and consider the establishment of “good data practice spot checks” to ensure that current practices adhere to guidelines established by the organization and community best practices. Organizations with significant investments in specific research domains can consider creating roles for Chief Information Officers or Chief Data Officers to augment existing governance structures. Finally, institutions can actively participate in organizations that support the development and adoption of data standards, especially in areas relevant to their research portfolio. These include domain-specific standards development organizations such as Health Level Seven International [32], Observational Health Data Sciences and Informatics (OHDSI) [33], and the Global Alliance for Genomics and Health (GA4GH) [34], as well as communities dedicated to advancing the creation and dissemination of shareable research outputs, such as Mobilizing Computable Biomedical Knowledge [35].

**Box 6. Data quality research workforce questions**

- Where can I find resources to help me understand data quality?
- Can I pay for consultation or curation services to help my lab with this work?
- What license should I apply to my data?
- Are there minimal information standards for the experiments my lab is conducting?

## Rule 10: Incorporate and refine data ethics frameworks and practices

Data sharing and data ethics are inextricably linked. Organizations should intentionally and continuously engage ethical concerns and principles to support data sharing and address systemic biases and injustices. The Federal Data Strategy’s Data Ethics Framework defines data ethics as “the norms of behavior that promote appropriate judgments and accountability when acquiring, managing, or using data, with the goals of protecting civil liberties, minimizing risks to individuals and society, and maximizing the public good” [36]. As with data quality and reuse best practices, organizations should address ethical issues early and throughout data sharing processes and can use governance, infrastructure, and training to do so.

A critical framework to consider is the CARE Principles for Indigenous Data Governance (Collective benefit, Authority to control, Responsibility, and Ethics) [37]. By operationalizing the CARE Principles with FAIR, data sovereignty rights can be supported and asserted through machine actionability, integrating a focus on people and purpose, and resolving Indigenous Peoples’ rights to and interests in their data [38]. Additionally, the Federal Data Strategy’s Data Ethics Framework provides a set of Data Ethics Tenets that can serve as a reference point for addressing local aspects of data sharing and guide accountability [36].

**Box 7. Ethics research workforce questions**

- Where can I learn more about how to implement ethical data practices?
- Besides privacy, what do I need to consider when developing my data sharing plan?
- Does the organization have resources in place to support CARE and Data Sovereignty?
- How do community research partners inform the organization’s data sharing practices and policies?

## Final thoughts

With these 10 simple rules, we intend to provide organizations with a landscape understanding of the factors involved in supporting data sharing to help guide strategic planning to grow organizational data sharing capacity. This work was created with several considerations in mind. First, the rules and environmental characteristics they describe are interdependent. It is difficult to advance maturity in one domain without addressing strengths and weaknesses in another. Second, the Research Data Sharing Capacity Maturity Model and the levels of maturity it defines are aspirational and set goals for organizations to strive towards in their advancement. As noted above, research institutions are at various stages of their local journey, and this framework aids in planning improvement initiatives.

The reality of the current moment finds many institutions in the earliest stages of considering what services, resources, and infrastructure they should implement to support data sharing. We aim to help organizations cultivate a cohesive approach to data sharing and shape the development of policies, infrastructure, and institutional values that support data sharing for the common good, not just compliance. Every member of an organization has a role to play in these conversations. This paper and the “Research Data Sharing: A Maturity Model for Organizational Capacity” provide a framework to coordinate practical communication and action across roles and responsibilities at the individual, group, and organizational levels.

The authors of this paper typically work in the biomedical research environment, which requires specific security, privacy, and ethical considerations to meet data sharing requirements for human participant research. While we reference aspects of this context in this paper and also note them in our maturity model, we have not addressed the details of this biomedical context with the hope that other discipline communities will expand and refine the recommendations and model we have developed to reflect the specific and sometimes different issues they must tackle to support data sharing. Thus, we invite the broader data sharing community to review, critique, and adapt “Research Data Sharing: A Maturity Model for Organizational Capacity”.

## References

[1] NOT-OD-21-013: Final NIH Policy for Data Management and Sharing [Internet]. [cited 2022 Oct 16]. Available from: https://grants.nih.gov/grants/guide/notice-files/NOT-OD-21-013.html.

[2] Gonzales S, Carson MB, Holmes K. Ten simple rules for maximizing the recommendations of the NIH data management and sharing plan. PLoS Comput Biol. 2022 Aug 3;18(8):e1010397. doi: 10.1371/journal.pcbi.1010397 35921268 PMC9348704

[3] Michener WK. Ten Simple Rules for Creating a Good Data Management Plan. PLoS Comput Biol. 2015 Oct 22;11(10):e1004525. doi: 10.1371/journal.pcbi.1004525 26492633 PMC4619636

[4] Champieux RE, Phuong J, Dorr D, Holmes K, Rojevsky S, Solomonides A, et al. Research Data Sharing: A Maturity Model for Organizational Capacity. 2022 Nov 27 [cited 2022 Nov 27]. Available from: https://zenodo.org/record/7369811.

[5] Crowston K, Qin J. A capability maturity model for scientific data management: Evidence from the literature. Proc Am Soc Inf Sci Technol. 2011;48(1):1–9.

[6] Knosp BM, Barnett WK, Anderson NR, Embi PJ. Research IT maturity models for academic health centers: Early development and initial evaluation. J Clin Transl Sci. 2018 Oct;2(5):289–94. doi: 10.1017/cts.2018.339 30828469 PMC6390403

[7] CTS Personas [Internet]. CTS Personas. 2023 [cited 2023 Mar 22]. Available from: https://zenodo.org/communities/cts-personas.

[8] User story. In: Wikipedia [Internet]. 2023 [cited 2023 Mar 23]. Available from: https://en.wikipedia.org/w/index.php?title=User_story&oldid=1145675576.

[9] Inclusive Team Science Toolkit–CREDITS [Internet]. [cited 2023 Mar 22]. Available from: https://credits.ucsb.edu/inclusive-team-science-toolkit/.

[10] Spring B, Klyachko E, Rak P, McFadden H, Hedeker D, Siddique J, et al. Online, cross-disciplinary team science training for health and medical professionals: Evaluation of COALESCE (teamscience.net). J Clin Transl Sci. 2019 Jul 18;3(2–3):82–9. doi: 10.1017/cts.2019.383 31660230 PMC6802413

[11] Lerner D, Palm ME, Concannon TW, editors. Broadly Engaged Team Science in Clinical and Translational Research [Internet]. Cham: Springer International Publishing; 2022 [cited 2023 Mar 22]. Available from: https://link.springer.com/10.1007/978-3-030-83028-1.

[12] Home | Research Data Management Service Group [Internet]. [cited 2023 Mar 22]. Available from: https://data.research.cornell.edu/.

[13] Research Data Management @Harvard [Internet]. [cited 2023 Mar 22]. Available from: https://researchdatamanagement.harvard.edu/home.

[14] Jenkins D. Introduction to Data Sharing and Integration [Internet]. 2020 [cited 2022 Oct 16]. Available from: https://aisp.upenn.edu/aisp-intro/.

[15] National Academies of Sciences E. Open Science by Design: Realizing a Vision for 21st Century Research [Internet]. 2018 [cited 2022 Oct 16]. Available from: https://nap.nationalacademies.org/catalog/25116/open-science-by-design-realizing-a-vision-for-21st-century. 30212065

[16] Tenopir C, Palmer CL, Metzer L, van der Hoeven J, Malone J. Sharing data: Practices, barriers, and incentives. Proc Am Soc Inf Sci Technol. 2011;48(1):1–4.

[17] Piwowar HA, Becich MJ, Bilofsky H, Crowley RS. Towards a Data Sharing Culture: Recommendations for Leadership from Academic Health Centers. PLoS Med. 2008 Sep;5(9):e183. doi: 10.1371/journal.pmed.0050183 18767901 PMC2528049

[18] Wood-Charlson EM, Crockett Z, Erdmann C, Arkin AP, Robinson CB. Ten simple rules for getting and giving credit for data. PLoS Comput Biol. 2022 Sep 29;18(9):e1010476. doi: 10.1371/journal.pcbi.1010476 36173960 PMC9521804

[19] Vierkant P. Wellcome Trust and the Chan Zuckerberg Initiative Partner with DataCite to Build the Open Global Data Citation Corpus [Internet]. DataCite Blog. 2023 [cited 2023 Mar 22]. Available from: https://blog.datacite.org/data-citation-corpus-announcement-2023/.

[20] Harvard Dataverse [Internet]. [cited 2022 Oct 22]. Available from: https://dataverse.harvard.edu/.

[21] InvenioRDM—inveniosoftware.org [Internet]. [cited 2022 Oct 22]. Available from: https://inveniosoftware.org/products/rdm/.

[22] Zenodo—Research. Shared. [Internet]. [cited 2022 Oct 22]. Available from: https://zenodo.org/.

[23] Dryad Our Mission [Internet]. [cited 2022 Oct 22]. Available from: https://datadryad.org/stash/our_mission.

[24] The Carpentries [Internet]. The Carpentries. [cited 2022 Oct 16]. Available from: https://carpentries.org/index.html.

[25] DataWorks! [Internet]. [cited 2022 Oct 16]. Available from: https://www.faseb.org/resources/data-science-and-informatics/dataworks.

[26] NNLM National Center for Data Services (NCDS) [Internet]. [cited 2022 Oct 16]. Available from: https://www.nnlm.gov/about/centers/ncds.

[27] Wilkinson MD, Dumontier M, Aalbersberg IjJ, Appleton G, Axton M, Baak A, et al. The FAIR Guiding Principles for scientific data management and stewardship. Sci Data. 2016 Mar 15;3(1):160018. doi: 10.1038/sdata.2016.18 26978244 PMC4792175

[28] NIH Common Data Elements (CDE) Repository [Internet]. [cited 2022 Oct 22]. Available from: https://cde.nlm.nih.gov/home.

[29] DataCite Schema [Internet]. DataCite Schema. [cited 2022 Oct 22]. Available from: http://schema.datacite.org/.

[30] Dataset Search [Internet]. [cited 2023 Mar 22]. Available from: https://datasetsearch.research.google.com/.

[31] DataCite Search [Internet]. [cited 2022 Oct 22]. Available from: https://search.datacite.org/.

[32] Health Level Seven International—Homepage | HL7 International [Internet]. [cited 2022 Oct 16]. Available from: https://www.hl7.org/.

[33] OHDSI–Observational Health Data Sciences and Informatics [Internet]. [cited 2022 Oct 16]. Available from: https://www.ohdsi.org/.

[34] Global Alliance for Genomics and Health (GA4GH) [Internet]. [cited 2022 Nov 27]. Available from: https://www.ga4gh.org/.

[35] Mobilizing Computable Biomedical Knowledge [Internet]. [cited 2022 Oct 16]. Available from: https://mobilizecbk.med.umich.edu/.

[36] Data Ethics Framework [Internet]. [cited 2022 Oct 16]. Available from: https://strategy.data.gov/assets/docs/2020-federal-data-strategy-framework.pdf.

[37] Carroll SR, Garba I, Figueroa-Rodríguez OL, Holbrook J, Lovett R, Materechera S, et al. The CARE Principles for Indigenous Data Governance. Data Sci J. 2020 Nov 4;19(1):43.

[38] Carroll SR, Herczog E, Hudson M, Russell K, Stall S. Operationalizing the CARE and FAIR Principles for Indigenous data futures. Sci Data. 2021 Apr 16;8(1):108. doi: 10.1038/s41597-021-00892-0 33863927 PMC8052430

